# Incidence and Predictors of Acute Kidney Injury Following Tricuspid Valve Surgery: The Prognostic Value of Right Ventricular Length–Force Relationship

**DOI:** 10.3390/jcm15135155

**Published:** 2026-07-02

**Authors:** Sercan Tak, Özant Helvacı, Erkan İriz, Hikmet Selçuk Gedik, Mustafa Hakan Zor, Abdullah Özer, Başak Koçak, Yonca Durkan, Taha Enes Çetin, Gürsel Levent Oktar

**Affiliations:** 1 Department of Cardiovascular Surgery, Faculty of Medicine, Gazi University, Ankara 06560, Turkey; erkaniriz@gazi.edu.tr (E.İ.); hsgedik@gazi.edu.tr (H.S.G.); basakkocak@gazi.edu.tr (B.K.);; 2 Department of Nephrology, Faculty of Medicine, Gazi University, Ankara 06560, Turkey; ozanthelvaci@gazi.edu.tr (Ö.H.);

**Keywords:** tricuspid valve surgery, acute kidney injury, right ventricle, systolic pulmonary artery pressure, tricuspid annular plane systolic excursion

## Abstract

**Background/Objectives**: Tricuspid valve surgery carries a high risk of postoperative acute kidney injury (AKI) due to pre-existing right ventricular dysfunction and congestive end-organ remodeling. We aimed to evaluate the incidence and predictors of postoperative AKI, with particular focus on the prognostic value of the TAPSE/sPAP index. **Methods**: This retrospective, single-center study evaluated adult patients who underwent tricuspid valve surgery (isolated/concomitant) between 2010 and 2025. The primary outcome was postoperative AKI of any stage, defined by the Kidney Disease: Improving Global Outcomes (KDIGO) criteria. Predictors were identified using a pre-specified multivariable logistic regression model including baseline estimated glomerular filtration rate (eGFR), cardiopulmonary bypass time, and EuroSCORE II; TAPSE/sPAP associations with severe renal outcomes were assessed univariably and presented as exploratory. **Results**: Of 80 patients, postoperative AKI occurred in 32 (40.0%), with 16 (20.0%) requiring renal replacement therapy. The pre-specified multivariable model discriminated any-stage AKI (AUC 0.86, 95% CI 0.77–0.94). Lower baseline eGFR (adjusted OR 1.82 per 10 mL/min/1.73 m^2^ decrease, *p* < 0.001) and higher EuroSCORE II (adjusted OR 1.53 per point, *p* = 0.03) were independent predictors. The TAPSE/sPAP index was not associated with any-stage AKI (*p* = 0.42), but lower values predicted advanced renal outcomes, including KDIGO stage ≥ 2 AKI (OR 2.11, *p* = 0.03) and the requirement for renal replacement therapy (OR 2.71, *p* = 0.01). Outcomes did not differ between isolated (*n* =14) and combined procedures (*n* = 66; AKI 35.7% vs. 40.9%, *p* = 0.77). **Conclusions**: Lower preoperative eGFR and higher EuroSCORE II independently predict any-stage postoperative AKI. In univariable analysis, the TAPSE/sPAP index identified the subgroup with severe renal outcomes; this exploratory finding requires prospective validation. Whether perioperative renal protection depends on addressing right-sided filling pressures rather than augmenting forward flow alone requires prospective testing.

## 1. Introduction

Acute kidney injury (AKI) is among the most critical complications of cardiac surgery, with a reported incidence of 5% to 50.6% [1,2]. Under the comprehensive Kidney Disease: Improving Global Outcomes (KDIGO) criteria, which capture subtle changes in serum creatinine (SCr) and urine output, incidence may reach 60% to 70% [3,4]. Postoperative AKI is a well-established independent risk factor for short- and long-term mortality, prolonged intensive care unit (ICU) and hospital stays, and complications such as major infections [5]. Although most cases are mild and the requirement for renal replacement therapy (RRT) is limited to approximately 1% to 2% [6], even a transient episode predisposes patients to progressive chronic kidney disease and cardiovascular events [7].

Beyond conventional patient-related factors, cardiac surgery entails unique perioperative stressors, including prolonged cardiopulmonary bypass (CPB) and aortic cross-clamp times, ischemia–reperfusion injury, systemic inflammation, and oxidative stress [8]. Confounders that directly precipitate renal injury—massive hemorrhage, blood product transfusions, perioperative anemia, nephrotoxic agents, and high-dose vasopressor support—frequently cluster perioperatively [9]. Preoperative cardiac status is another key driver; patients with left ventricular (LV) dysfunction face a heightened risk of renal ischemia due to diminished systemic perfusion pressures [10].

Independent of these mechanisms, venous congestion from tricuspid regurgitation (TR) has emerged as a distinct driver of renal dysfunction [11]. Elevated central venous pressure (CVP) secondary to TR translates directly into renal venous hypertension, attenuating the transrenal pressure gradient between the renal artery and renal vein and thereby severely reducing the glomerular filtration rate [11]. This intrarenal congestion and subsequent edema also mechanically compress the renal tubules and capillary microcirculation, causing localized ischemia and direct nephron loss [12]. Compounding this, the high-dose diuretic regimens mandated preoperatively for volume management further contribute to AKI. Consequently, tricuspid valve surgery (TVS) carries a high risk, particularly in patients who have already developed right ventricular (RV) dysfunction and congestive end-organ damage. In this context, echocardiographic indicators of RV afterload and longitudinal function—systolic pulmonary artery pressure (sPAP) and tricuspid annular plane systolic excursion (TAPSE)—reflect the severity of chronic renal venous congestion and impaired forward flow [13]. However, recent evidence underscores that intervention before advanced congestive organ remodeling yields significantly superior outcomes [14].

Although the detrimental effects of venous hypertension are well recognized, contemporary data on the prognostic value of preoperative RV metrics (sPAP and TAPSE) for AKI following TVS remain scarce and inconsistent. This study aims to evaluate the incidence and prognostic risk factors of postoperative AKI in patients undergoing TVS, performed either as an isolated or concomitant procedure, with a specific focus on preoperative echocardiographic and clinical predictors.

## 2. Materials and Methods

### 2.1. Study Population and Design

This was a retrospective, single-center observational study conducted in accordance with the principles of the Declaration of Helsinki. The study protocol was reviewed and formally approved by our Institutional Ethics Committee (Approval No: E.1472965). We screened the institutional medical database for all consecutive adult patients who underwent TVS due to TR at our center between January 2010 and June 2025 to minimize selection bias. Pediatric patients operated on due to congenital heart anomalies were excluded. Patients with pre-existing end-stage renal disease requiring chronic maintenance hemodialysis, and those who died intraoperatively or within the first 24 h postoperatively (to exclude early non-renal mortality), were also excluded. The remaining patients with complete perioperative data were included in the final cohort.

### 2.2. Data Collection and Definitions of Outcomes

Patient data were retrieved from the institution’s electronic health records and standardized clinical charts. Preoperative baseline characteristics included demographic data, medical history, comorbidities, and functional capacity graded according to the New York Heart Association (NYHA) classification. Baseline laboratory parameters, obtained at the closest time point prior to surgery, included hemoglobin (Hb), SCr, estimated glomerular filtration rate (eGFR), and N-terminal pro-B-type natriuretic peptide (NT-proBNP). Preoperative transthoracic echocardiographic assessments provided metrics for left ventricular ejection fraction (LVEF), TAPSE, and sPAP. The derived TAPSE/sPAP ratio and EuroSCORE II were calculated for each patient. Intraoperative variables included total CPB time, aortic cross-clamp time, and the lowest hematocrit (Hct) recorded during CPB. Postoperative courses were monitored for total ICU stay, total hospital stay, and mechanical ventilation duration. Chronic kidney disease was defined according to KDIGO criteria as eGFR < 60 mL/min/1.73 m^2^ persisting for at least three months or a documented pre-existing diagnosis. Postoperative SCr was routinely measured in all patients within the first 6 h following surgery; subsequent assessments were tailored based on kinetic trends, with SCr monitored at 12 h intervals in patients exhibiting an upward trajectory and at 24 h intervals for those with stable values, thereby providing temporal resolution sufficient for KDIGO staging. Additionally, hourly urine output was prospectively recorded in the cardiovascular ICU, allowing application of both SCr and urine output criteria. Nephrotoxic medications were withheld during the perioperative period as a matter of institutional policy. Vasopressor and inotrope use was guided by hemodynamic need at the attending team’s discretion, and packed red cell transfusions were administered as clinically indicated; the lowest intraoperative Hct served as a proxy for hemodilution and transfusion exposure.

The primary outcome was postoperative AKI of any stage, defined and staged according to the KDIGO classification. Secondary outcomes were major postoperative adverse events, including low cardiac output syndrome (LCOS), requirement for intra-aortic balloon pump (IABP) or extracorporeal membrane oxygenation (ECMO) support, new-onset atrial fibrillation (AF), stroke, sepsis, requirement for postoperative RRT, pulmonary complications, reoperation due to bleeding, and in-hospital mortality.

### 2.3. Surgical Management

All surgical interventions were performed via a standard median sternotomy. CPB was established using standard ascending aortic and bicaval cannulation. Except for a subgroup of patients undergoing isolated tricuspid valve replacement (TVR) on a beating heart with total CPB support, all operations were executed under aortic cross-clamping with myocardial protection achieved via cold blood cardioplegia. Surgical techniques utilized for the tricuspid valve intervention consisted of either TVR (bioprosthetic or mechanical) or tricuspid valve repair using ring annuloplasty or the De Vega annuloplasty technique.

### 2.4. Special Definitions

#### 2.4.1. The TAPSE/sPAP Index

The index derived from TAPSE and sPAP has emerged as a surrogate marker of the RV longitudinal function-to-developed-pressure relationship. As previously validated in heart failure cohorts, the relationship between TAPSE and sPAP serves as a non-invasive index of the length–force relationship and is not influenced by the severity of concomitant LV dysfunction [15].

#### 2.4.2. AKI Staging (KDIGO)

Postoperative AKI was defined and classified into three severity stages based on the KDIGO clinical practice guidelines. Staging was determined by tracking changes in SCr relative to baseline and/or weight-adjusted urine output, as detailed in Table 1.

#### 2.4.3. Indications for Renal Replacement Therapy

Renal replacement therapy was initiated using an early-initiation strategy modeled on the ELAIN trial protocol [16]. Patients reaching KDIGO stage 3 AKI were considered for RRT, and those at KDIGO stage 2 were started when classic indications were present (refractory hyperkalemia, metabolic acidosis unresponsive to medical therapy, uremic complications, or hypervolemia not controlled by diuretics). The 20% RRT incidence in this cohort reflects this early-initiation policy combined with the unfavorable baseline renal profile of patients undergoing TVS, of whom 43.8% had chronic kidney disease at baseline.

### 2.5. Statistical Analysis

Statistical analyses were performed using IBM SPSS Statistics for Windows, Version 25.0 (IBM Corp., Armonk, NY, USA). Normality of continuous variables was assessed using both visual (histograms, probability plots) and analytical methods (Kolmogorov–Smirnov test). Continuous data are expressed as mean ± standard deviation (SD) for normally distributed variables, and as median with interquartile range (IQR, 25th–75th percentiles) for non-normally distributed variables. Categorical variables are reported as frequencies and percentages (*n*, %). Differences in continuous variables between patients who developed AKI and those who did not were analyzed using the independent Student’s *t*-test or the Mann–Whitney U test, as appropriate. Categorical data and proportions were compared using the Chi-square test or Fisher’s exact test. To identify predictors of postoperative AKI, univariable logistic regression analysis was initially performed. A pre-specified multivariable logistic regression model was constructed using the Enter method, incorporating baseline eGFR, CPB time, and EuroSCORE II to determine adjusted odds ratios (OR) and 95% confidence intervals (CI). To prevent overparameterization and avoid model overfitting, the construction of the multivariable logistic regression model was strictly guided by the mathematical constraint of outcome events. Adhering to the established rule of requiring approximately 10 events per predictor variable, the multivariable analysis was restricted to three pre-specified clinical covariates based on our 32 postoperative AKI events. Due to this limited number of events for advanced renal outcomes, the association of the TAPSE/sPAP index with KDIGO stage ≥ 2 AKI and the need for RRT was evaluated using univariable logistic regression to avoid model overfitting. The discriminative performance of the pre-specified multivariable model, standalone echocardiographic parameters (TAPSE, sPAP), and the TAPSE/sPAP index across different severity thresholds of AKI was evaluated using Receiver Operating Characteristic (ROC) curve analysis. The areas under the ROC curve (AUC) with corresponding 95% CIs were calculated. A two-sided *p*-value of <0.05 was considered statistically significant for all analyses. Multicollinearity among predictors of the pre-specified multivariable model was assessed using variance inflation factors and the Spearman correlation between EuroSCORE II and baseline eGFR; a sensitivity multivariable model excluding eGFR was additionally fitted to verify that the EuroSCORE II effect was not driven by collinearity. Given the limited cohort size and the modest AUCs observed, the discriminative performance of the multivariable model and of the TAPSE/sPAP index is interpreted as modest-to-moderate rather than as evidence of strong predictive performance.

Incremental discrimination of the TAPSE/sPAP index was assessed by DeLong’s test, continuous net reclassification improvement (NRI), and integrated discrimination improvement (IDI). For any-stage AKI, the pre-specified model (eGFR + CPB + EuroSCORE II) served as the reference. For KDIGO stage ≥ 2 AKI and the need for RRT, the limited event counts (*n* = 19 and *n* = 16) precluded a three-variable model, so the reference was baseline eGFR alone.

## 3. Results

### 3.1. Patient Characteristics

A total of 80 patients undergoing cardiac surgery involving the tricuspid valve under CPB were included. The mean age was 61.5 ± 12.3 years and 60 patients (75.0%) were female. Hypertension was present in 58 patients (72.5%), diabetes mellitus in 23 (28.7%) and AF in 63 (78.8%). Chronic kidney disease was present in 35 (43.8%) patients. The mean eGFR was 84.6 ± 23.1 mL/min/1.73 m^2^, and the median EuroSCORE II was 4 (IQR, 2–5). The mean CPB time was 148.5 ± 56.3 min and the mean cross-clamp time was 115.3 ± 38.4 min. Baseline characteristics of the AKI and non-AKI cohorts are presented in Table 2. A history of previous cardiac surgery was present in 23 (28.7%) patients. Isolated TVS was performed in 14 (17.5%) patients, whereas 66 (82.5%) patients underwent concomitant cardiac procedures. Regarding the specific valvular interventions, 22 (27.5%) patients underwent TVR, while the remaining patients (72.5%) received either De Vega or ring annuloplasty. The surgical procedures performed are detailed in Table 3.

### 3.2. Postoperative AKI and Other Secondary Outcomes

Postoperative AKI occurred in 32 patients (40.0%). KDIGO stages 1, 2 and 3 AKI were observed in 13 (16.3%), 9 (11.3%) and 10 (12.5%) patients, respectively. RRT was required in 16 patients (20.0%). In-hospital mortality occurred in 15 (18.8%) patients. Regarding postoperative complications, an IABP and ECMO were required in five (6.3%) patients each, while reoperation due to bleeding was performed in four (5.0%) cases. Additionally, stroke was observed in two (2.5%) patients, new-onset AF in seven (8.8%), and sepsis in nine (11.3%). Respiratory complications developed in 25 (31.3%) patients, with 21 of them requiring re-intubation.

When stratified by surgical approach (isolated TVS, *n* = 14; combined procedures, *n* = 66), any-stage AKI occurred in 5/14 (35.7%) and 27/66 (40.9%) respectively (*p* = 0.77), with no significant differences in KDIGO stage ≥ 2 AKI (35.7% vs. 21.2%; *p* = 0.30), the need for RRT (21.4% vs. 19.7%; *p* = 1.00) or in-hospital mortality (21.4% vs. 18.2%; *p* = 0.97). Patients undergoing isolated TVS more often had a history of previous cardiac surgery (10/14, 71.4% vs. 13/66, 19.7%; *p* < 0.001). The full subgroup comparison, including intraoperative variables, is presented in Table 4.

### 3.3. Comparison of Patients with and Without AKI

Compared with patients without AKI, those who developed AKI were older and had a higher prevalence of hypertension, chronic kidney disease, and higher preoperative SCr and lower eGFR, together with higher EuroSCORE II values (all *p* < 0.001). On echocardiography, TAPSE was lower in the AKI group (*p* = 0.008), whereas sPAP and the TAPSE/sPAP index did not differ between groups. The lowest intraoperative Hct was also lower in patients with AKI (*p* = 0.002). Full comparisons are provided in Table 2.

### 3.4. Predictors of Postoperative AKI

In a univariable logistic regression, age, hypertension, chronic kidney disease, NYHA class III–IV, eGFR, TAPSE, EuroSCORE II, and the lowest intraoperative Hct were associated with postoperative AKI (Table 5). Diabetes mellitus, AF, NT-proBNP, LVEF, the TAPSE/sPAP index and CPB time did not reach statistical significance in the univariable analysis.

**Figure 1:**
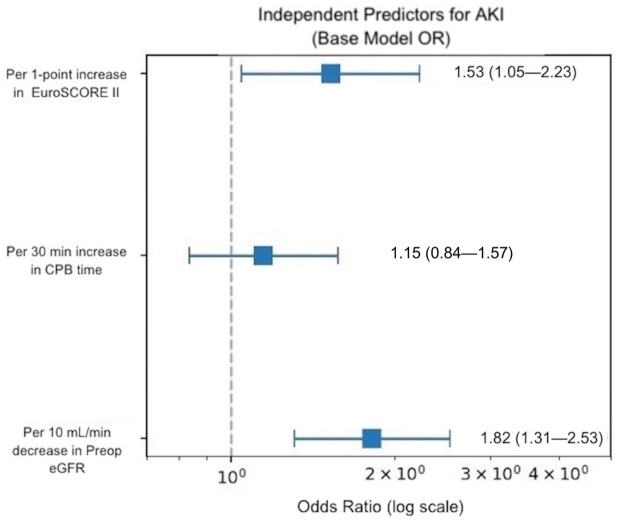
Forest plot of adjusted ORs from the pre-specified multivariable logistic regression model for postoperative AKI. ORs are expressed per clinically meaningful unit of change (10 mL/min/1.73 m^2^ decrease in eGFR, 30 min increase in CPB, 1-point increase in EuroSCORE II). Horizontal bars indicate 95% confidence intervals; the vertical dotted line represents the null effect (OR = 1). Numerical estimates are reported next to each point. Abbreviations: AKI, acute kidney injury; CPB, cardiopulmonary bypass; eGFR, estimated glomerular filtration rate; OR, odds ratio.

**Figure 2:**
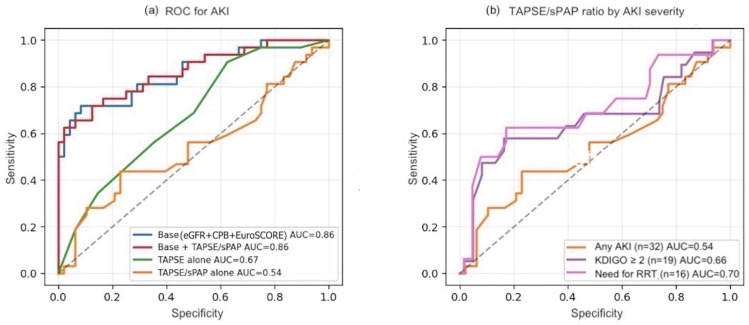
ROC curve analysis of the TAPSE/sPAP index and multivariable models for predicting postoperative acute kidney injury and its severity. ROC curves for the prediction of any postoperative AKI (**a**). Curves are shown for the pre-specified multivariable model (eGFR, CPB time and EuroSCORE II), the same model after addition of the TAPSE/sPAP index, TAPSE alone and the TAPSE/sPAP index alone. AUCs are indicated in the legend. ROC curves of the TAPSE/sPAP index across renal outcomes of increasing severity (**b**): Any AKI (*n* = 32), KDIGO stage ≥ 2 (*n* = 19) and the need for RRT (*n* = 16). The diagonal dashed line represents the reference line of random chance (AUC = 0.50). Abbreviations: ROC, receiver operating characteristic; AUC, area under the curve; AKI, acute kidney injury; CPB, cardiopulmonary bypass; eGFR, estimated glomerular filtration rate; TAPSE, tricuspid annular plane systolic excursion; sPAP, systolic pulmonary artery pressure; KDIGO, Kidney Disease Improving Global Outcomes; RRT, renal replacement therapy.

A pre-specified multivariable logistic regression model including eGFR, CPB time and EuroSCORE II (corresponding to 10.7 events per variable) identified lower eGFR (adjusted OR 1.82 per 10 mL/min/1.73 m^2^ decrease; 95% CI 1.31–2.53; *p* < 0.001) and higher EuroSCORE II (adjusted OR 1.53 per 1 point; 95% CI 1.05–2.23; *p* = 0.03) as independent predictors of postoperative AKI. CPB time was not independently associated with AKI in the adjusted model (adjusted OR 1.15 per 30 min increase; 95% CI 0.84–1.57; *p* = 0.39) (Figure 1). The model demonstrated an AUC of 0.86 (95% CI 0.77–0.94) (Figure 2a).

Multicollinearity among the three pre-specified predictors was not problematic: variance inflation factors were 1.33 for eGFR, 1.05 for CPB time, and 1.39 for EuroSCORE II (all < 2). The Spearman correlation between EuroSCORE II and baseline eGFR was moderate (rho = −0.53, *p* < 0.001), reflecting the partial inclusion of renal function within the EuroSCORE II algorithm but not at a degree that compromised independent estimation. In a sensitivity multivariable model excluding eGFR, EuroSCORE II remained an independent predictor of postoperative AKI (adjusted OR 1.88 per 1-point increase; 95% CI 1.35–2.62; *p* < 0.001), supporting that its effect was not solely a proxy for baseline renal function.

### 3.5. Association of the TAPSE/sPAP Index with Renal Outcomes of Increasing Severity

When examined across renal outcomes of increasing severity, the TAPSE/sPAP index was not associated with any-stage AKI (*p* = 0.42) (Figure 2a), but lower values were associated with KDIGO stage ≥ 2 AKI (OR 2.11 per 0.1 decrease; *p* = 0.03) and with the need for RRT (OR 2.71 per 0.1 decrease; *p* = 0.01) (Figure 2b). The discriminative ability of the index increased progressively with outcome severity, as shown in Table 6.

Adding the TAPSE/sPAP index to the pre-specified model did not improve discrimination of any-stage AKI (DeLong *p* = 0.32; cNRI *p* = 0.52; IDI *p* = 0.32). Against an eGFR-only reference, lower TAPSE/sPAP values remained independently associated with KDIGO stage ≥ 2 AKI (adjusted OR 2.23, 95% CI 1.09–4.57, *p* = 0.028; cNRI *p* = 0.05; IDI *p* = 0.10) and the need for RRT (adjusted OR 2.92, 95% CI 1.30–6.55, *p* = 0.009; cNRI *p* = 0.007; IDI *p* = 0.010); DeLong AUC comparisons were not significant (*p* = 0.35 and *p* = 0.31), as expected at this sample size.

## 4. Discussion

Postoperative AKI following cardiac surgery is a major complication associated with adverse outcomes and increased mortality. Although cardiac surgery-associated AKI has been studied extensively, its risk factors and stage-specific incidence in homogeneous TVS cohorts have not been comprehensively evaluated, and subgroup analyses suggest that TVS may carry a higher AKI risk than certain other procedures [3,17]. To our knowledge, this is one of the few contemporary studies demonstrating a direct relationship between the preoperative RV length–force relationship and the severity gradient of postoperative renal dysfunction in this population. Our principal finding is that lower baseline eGFR and higher EuroSCORE II are independent predictors of any-stage AKI, whereas the TAPSE/sPAP index showed univariable associations only with severe renal outcomes (KDIGO stage ≥ 2 AKI and the need for RRT) rather than with any-stage AKI; given the small number of severe events, this finding requires prospective validation.

In our cohort, the incidence of postoperative AKI was 40%, higher than typically reported after general cardiac surgery. In one of the initial large-scale studies of renal outcomes after TVS, Englberger et al. reported an incidence of 30% [18]. Our higher incidence likely reflects the use of contemporary KDIGO criteria, which incorporate both SCr and urine output and thus capture milder injury; in cohorts where KDIGO criteria were fully applied, AKI incidence has reached 60% to 70.5% [3,4].

Our pre-specified multivariable model combining preoperative eGFR, EuroSCORE II, and CPB time discriminated postoperative AKI well, with lower eGFR and higher EuroSCORE II as independent predictors. The central role of baseline eGFR in postoperative renal impairment is well recognized [19,20]. Although EuroSCORE II was designed to estimate perioperative mortality [21], validation studies confirm that high scores carry an increased AKI risk due to narrowed functional reserves [3,20]. Consistently, Machado et al. noted that elevated preoperative SCr and high EuroSCORE values, clustered with prolonged CPB, constitute the primary risk constellation for cardiac surgery-associated AKI [22].

Prolonged CPB is a well-established predictor of post-cardiac surgery renal injury via cumulative hemolysis, systemic inflammation, and ischemia–reperfusion injury [23]. Despite this, CPB time was not independently associated with AKI in our adjusted model (*p* = 0.39). This likely reflects the homogeneity of our cohort—most patients (82.5%) underwent concomitant complex procedures, narrowing CPB durations into a high, similar range across groups—compounded by limited power from the modest sample size. In this setting, patient-specific factors such as preoperative eGFR and EuroSCORE II appear more dominant than procedural duration in driving AKI.

TAPSE was significantly lower in patients who developed any-stage AKI and was a univariable risk factor (*p* = 0.008), underscoring the importance of longitudinal RV systolic function in this population. The TAPSE/sPAP index, however, did not differ by any-stage AKI, because sPAP was comparable between groups (*p* = 0.68); the non-discriminating sPAP denominator diluted the predictive signal carried by TAPSE. By contrast, the index became significant as renal injury deepened, with lower values associated with KDIGO stage ≥ 2 AKI and the need for RRT. This severity-dependent pattern is consistent with, but does not prove, a congestion-mediated mechanism specific to tricuspid regurgitation; direct hemodynamic correlates (central venous pressure, renal venous Doppler) were unavailable in this retrospective cohort, so the mechanistic interpretation that follows remains hypothetical. Whereas traditional cardiorenal paradigms emphasized low cardiac output and impaired arterial perfusion from LV dysfunction, the principal driver of worsening renal function in decompensated heart failure is backward venous congestion and elevated CVP rather than reduced cardiac index [11,24]. In tricuspid regurgitation, RV dysfunction acutely exacerbates an already chronically elevated CVP. Because of the encapsulated, non-compliant structure of the kidney, this pressure is transmitted to the renal veins, raising intrarenal interstitial pressure, compressing the tubules, compromising peritubular capillary flow, and triggering tubulointerstitial hypoxia and injury. Since glomerular filtration depends on the transglomerular pressure gradient, rising venous pressure directly blunts filtration; experimental models confirm that elevated venous pressure reduces renal blood flow more than an equivalent fall in arterial pressure [12]. Consistently, Massoth et al. showed that perioperative CVP elevation (>10 mmHg) raised postoperative AKI risk from 7.5% to 43.3% [25].

In our cohort, transient congestion-independent prerenal mechanisms likely predominated in mild AKI, whereas sustained congestion-mediated tubular injury came to the forefront in severe cases progressing to RRT. At this stage, the altered RV length–force relationship outweighs the dilutional effect of sPAP in the denominator, rendering the index significant. This illustrates that ratio-based biomarkers are not inherently superior to their components across all endpoints; their utility depends on how closely they align with the pathophysiology of the targeted outcome. The tricuspid surgery population is an ideal model to test this hypothesis, as RV-PA uncoupling and congestive nephropathy are most pronounced in this setting.

To clarify this right heart–kidney interplay, prospective studies incorporating early postoperative CVP monitoring and renal venous Doppler ultrasonography are needed to confirm the predictive value of the TAPSE/sPAP index and assess its incorporation into established risk-stratification scores.

Only 14 patients (17.5%) underwent isolated TVS; the remaining 66 had concomitant valvular, coronary, or other cardiac procedures. AKI rates were nonetheless comparable between groups (35.7% vs. 40.9%; *p* = 0.77), suggesting that patient-level factors (baseline renal function and overall operative risk) outweighed the surgical approach itself, consistent with the broader cardiorenal continuum as previously suggested by Provenzano et al. [26].

Additionally, the predominance of female patients (75.0%) in our study population is consistent with the contemporary literature, where a high female incidence is frequently reported due to the specific demographic distribution of both rheumatic and advanced functional tricuspid regurgitation [14,27]. While this gender distribution aligns with established international registries and supports the external validity of our findings within the tricuspid surgical population, replication in multicenter cohorts with wider etiological variation remains warranted.

Several limitations warrant consideration. First, this is a single-center, retrospective study with a modest sample size. The relatively limited size of our cohort is primarily driven by the retrospective nature of the study over a 15-year period, where a strict consecutive sampling strategy was applied. A significant number of patients had to be excluded due to incomplete or non-standardized preoperative echocardiographic datasets, particularly regarding right ventricular quantification metrics such as TAPSE or sPAP. While ensuring data completeness and analytical integrity for the remaining 80 patients, this data dependency inherently introduces selection bias and limits the overall statistical power for advanced subgroup analyses. Furthermore, the limited number of events for advanced renal outcomes precluded multivariable adjustment for these specific endpoints, meaning that these exploratory associations require validation in larger cohorts. Second, direct measurements of venous congestion, such as perioperative CVP monitoring, renal venous Doppler ultrasonography, or intrarenal venous flow patterns, were not available in our dataset, so the proposed mechanism of congestion-mediated tubulointerstitial injury rests on established physiological reasoning. Third, detailed tricuspid regurgitation characteristics (primary versus secondary etiology, graded severity, and right ventricular structural and functional metrics beyond TAPSE and sPAP) were not uniformly available and could not be analyzed. Fourth, perioperative red cell transfusion volume and cumulative vasopressor dose were not systematically captured in the retrospective dataset; the lowest intraoperative Hct (which differed between AKI and non-AKI groups, *p* = 0.002) served as an indirect proxy, but residual confounding from unmeasured exposure cannot be excluded. Finally, the cohort was limited to TVS (isolated or concomitant), which may restrict generalizability to isolated mitral/aortic or CABG populations; this restriction, however, is also a strength, as the tricuspid setting is where RV-PA uncoupling and congestive nephropathy are most relevant for testing this hypothesis.

## 5. Conclusions

In conclusion, lower preoperative eGFR and higher EuroSCORE II are independent predictors of postoperative AKI in patients undergoing TVS. The TAPSE/sPAP index, an integrated measure of RV longitudinal function relative to afterload, was associated in univariable analysis with severe renal outcomes (KDIGO stage ≥ 2 AKI and the need for RRT) but not with any-stage AKI; this exploratory finding requires prospective validation. Whether perioperative renal protection in this setting depends on addressing right-sided filling pressures rather than augmenting forward flow alone requires prospective testing.

## Figures and Tables

**Table 1:** AKI staging—KDIGO.

AKI Stage	Serum Creatinine Criteria	Urine Output Criteria
1	SCr increase ≥ 0.3 mg/dL within 48 h or SCr increase 1.5–1.9 fold from baseline	<0.5 mL/kg/hr for 6 h
2	SCr increase 2.0–2.9 fold from baseline	<0.5 mL/kg/hr for 12 h
3	SCr increase ≥ 3.0 fold from baseline or SCr increase ≥ 4 mg/dL or Initiated on RRT	<0.3 mL/kg/hr for 24 h or Anuria for 12 h

AKI, acute kidney injury; KDIGO, Kidney Disease: Improving Global Outcomes; SCr, serum creatinine; RRT, renal replacement therapy.

**Table 2:** Baseline characteristics and operative variables by postoperative AKI status.

Variable	No AKI (*n* = 48)	AKI (*n* = 32)	*p*-Value
Age, years	57 (49–67)	69 (66–74)	<0.001
Female sex, *n* (%)	34 (70.8)	26 (81.3)	0.43
Body mass index, kg/m^2^	25.1 (23.4–27.7)	27.2 (23.4–29.4)	0.37
Hypertension, *n* (%)	30 (62.5)	28 (87.5)	0.03
Diabetes mellitus, *n* (%)	11 (22.9)	12 (37.5)	0.25
Atrial fibrillation, *n* (%)	35 (72.9)	28 (87.5)	0.20
Previous cardiac surgery, *n* (%)	16 (33.3)	7 (21.9)	0.39
Chronic kidney disease, *n* (%)	12 (25.0)	23 (71.9)	<0.001
NYHA class III–IV, *n* (%)	19 (39.6)	20 (62.5)	0.07
Hemoglobin, g/dL	12.5 ± 1.6	11.9 ± 1.8	0.11
Creatinine, mg/dL	0.78 (0.68–0.88)	1.00 (0.80–1.10)	<0.001
eGFR, mL/min/1.73 m^2^	95.2 ± 16.1	68.7 ± 23.0	<0.001
NT-proBNP, pg/mL	1679 (772–3033)	2941 (1395–3923)	0.07
Left ventricular EF, %	60 (55–62)	60 (55–60)	0.65
Systolic PAP, mmHg	55 (47–60)	52 (45–63)	0.68
TAPSE, mm	17.5 (16.0–19.3)	16.0 (15.0–18.0)	0.008
TAPSE/sPAP index	0.33 ± 0.08	0.32 ± 0.09	0.43
EuroSCORE II	3.0 (2.0–4.0)	5.0 (4.0–6.0)	<0.001
Combined valve + CABG, *n* (%)	4 (8.3)	6 (18.8)	0.19
CPB time, min	146 (114–169)	146 (118–197)	0.50
Lowest hematocrit, %	23.7 ± 3.5	21.3 ± 3.1	0.002

Data are mean ± SD, median (interquartile range), or *n* (%). Group comparisons used Student’s *t* test or Mann–Whitney U test for continuous variables, and the χ^2^ test or Fisher’s exact test for categorical variables, as appropriate. AKI, acute kidney injury; NYHA, New York Heart Association; eGFR, estimated glomerular filtration rate; NT-proBNP, N-terminal pro-B-type natriuretic peptide; EF, ejection fraction; PAP, pulmonary artery pressure; TAPSE, tricuspid annular plane systolic excursion; CABG, coronary artery bypass grafting; CPB, cardiopulmonary bypass.

**Table 3:** Surgical procedures performed in the study population.

Type of Surgery	Number of Patients
Isolated TV surgery, *n* (%)	14 (17.5%)
Single valve + TV surgery, *n* (%)	39 (48.8%)
Double valve + TV surgery, *n* (%)	9 (11.3%)
Other surgery + TV surgery, *n* (%) *	8 (10.0%)
Any valve (including TV) surgery + CABG, *n* (%)	10 (12.5%)

TV, tricuspid valve; CABG, coronary artery bypass grafting. * Other procedures performed concomitantly with TV surgery: Ascending aorta replacement (*n* = 2) and atrial septal defect repair (*n* = 6).

**Table 4:** Baseline characteristics, intraoperative variables and renal outcomes by surgical subgroup.

Variable	Isolated TVS (*n* = 14)	Combined Procedures (*n* = 66)	*p*-Value
**Baseline characteristics**			
Age, years (median, IQR)	62.5 (53.8–68.5)	65.5 (53.0–69.0)	0.78
Previous cardiac surgery, *n* (%)	10 (71.4)	13 (19.7)	<0.001
EuroSCORE II (median, IQR)	3.0 (3.0–5.8)	3.5 (2.0–5.0)	0.43
Preoperative eGFR, mL/min/1.73 m^2^ (median, IQR)	92.0 (82.5–102.0)	89.5 (66.0–101.0)	0.65
Hemoglobin, g/dL (median, IQR)	11.4 (9.9–12.3)	12.4 (11.6–13.5)	0.009
**Intraoperative variables**			
CPB time, min (median, IQR)	74.5 (61.8–112.2)	150.5 (127.8–184.0)	<0.001
Aortic cross-clamp, min (median, IQR)	74.5 (70.2–113.2)	109.5 (97.2–133.2)	0.073
Lowest intraoperative hematocrit, % (median, IQR)	21.2 (19.4–25.8)	22.5 (19.8–25.2)	0.55
**Renal and clinical outcomes**			
Any-stage AKI, *n* (%)	5 (35.7)	27 (40.9)	0.77
KDIGO stage ≥ 2 AKI, *n* (%)	5 (35.7)	14 (21.2)	0.30
RRT required, *n* (%)	3 (21.4)	13 (19.7)	1.00
In-hospital mortality, *n* (%)	3 (21.4)	12 (18.2)	0.97

Continuous variables: median (interquartile range), Mann–Whitney U test. Categorical variables: count (%), Fisher’s exact test. Combined procedures comprised tricuspid valve surgery performed concomitantly with any other valvular procedure (*n* = 56) or with valvular surgery plus coronary artery bypass grafting (*n* = 10). AKI, acute kidney injury; CPB, cardiopulmonary bypass; eGFR, estimated glomerular filtration rate; IQR, interquartile range; KDIGO, Kidney Disease: Improving Global Outcomes; RRT, renal replacement therapy; TVS, tricuspid valve surgery.

**Table 5:** Univariable and multivariable logistic regression for postoperative acute kidney injury.

Predictor	Univariable OR (95% CI)	*p*	Adjusted OR (95% CI)	*p*
Age, per 1-year increase	1.11 (1.05–1.17)	<0.001	—	—
Hypertension, yes vs. no	4.20 (1.27–13.94)	0.02	—	—
Diabetes mellitus, yes vs. no	2.02 (0.76–5.39)	0.16	—	—
Atrial fibrillation, yes vs. no	2.60 (0.76–8.86)	0.13	—	—
Chronic kidney disease, yes vs. no	7.67 (2.79–21.06)	<0.001	—	—
NYHA class III–IV, yes vs. no	2.54 (1.01–6.38)	0.05	—	—
eGFR, per 10 mL/min/1.73 m^2^ decrease	1.98 (1.45–2.71)	<0.001	1.82 (1.31–2.53)	<0.001
NT-proBNP, per 1000 pg/mL	1.16 (0.99–1.35)	0.06	—	—
Left ventricular EF, per 5% decrease	1.07 (0.76–1.52)	0.69	—	—
TAPSE, per 1 mm decrease	1.38 (1.09–1.76)	0.008	—	—
TAPSE/sPAP index, per 0.1 decrease	1.25 (0.73–2.13)	0.42	—	—
EuroSCORE II, per 1 point	1.90 (1.37–2.65)	<0.001	1.53 (1.05–2.23)	0.03
CPB time, per 30 min	1.19 (0.93–1.52)	0.16	1.15 (0.84–1.57)	0.39
Lowest hematocrit, per 1% decrease	1.25 (1.07–1.44)	0.004	—	—

Multivariable model was pre-specified to include three a priori predictors (eGFR, CPB time, and EuroSCORE II), corresponding to 10.7 events per variable. Remaining variables are shown for univariable comparison only. Model discrimination: Area under the receiver operating characteristic curve 0.86 (95% CI 0.77–0.94). NYHA, New York Heart Association; eGFR, estimated glomerular filtration rate; NT-proBNP, N-terminal pro-B-type natriuretic peptide; EF, ejection fraction; TAPSE, tricuspid annular plane systolic excursion; sPAP, systolic pulmonary artery pressure; CPB, cardiopulmonary bypass.

**Table 6:** Univariable association of the TAPSE/sPAP index with renal outcomes of increasing severity.

Outcome	Events	OR per 0.1 Decrease (95% CI)	AUC (95% CI)	*p*-Value
Any-stage AKI	32	1.25 (0.73–2.13)	0.54 (0.41–0.67)	0.42
KDIGO stage ≥ 2	19	2.11 (1.07–4.16)	0.66 (0.50–0.83)	0.03
Need for RRT	16	2.71 (1.26–5.84)	0.70 (0.53–0.86)	0.01

Logistic regression with the TAPSE/sPAP index modeled as a continuous variable; ORs are expressed per 0.1-unit decrease. TAPSE, tricuspid annular plane systolic excursion; sPAP, systolic pulmonary artery pressure; AUC, area under the curve; AKI, acute kidney injury; KDIGO, Kidney Disease Improving Global Outcomes; RRT, renal replacement therapy; OR, odds ratio.

## Data Availability

The data presented in this study are available on request from the corresponding author due to privacy and ethical restrictions regarding patient data.

## Abbreviations

AKI	acute kidney injury
KDIGO	Kidney Disease: Improving Global Outcomes
SCr	serum creatinine
RRT	renal replacement therapy
NYHA	New York Heart Association
eGFR	estimated glomerular filtration rate
NT-proBNP	N-terminal pro-B-type natriuretic peptide
EF	ejection fraction
sPAP	systolic pulmonary artery pressure
TAPSE	tricuspid annular plane systolic excursion
CABG	coronary artery bypass grafting
CPB	cardiopulmonary bypass
TVS	tricuspid valve surgery
CVP	central venous pressure
LV	left ventricle
RV	right ventricle
AF	atrial fibrillation
ECMO	extracorporeal membrane oxygenation
IABP	intra-aortic balloon pump
TVR	tricuspid valve replacement
